# Soybean knowledge base (SoyKB): a web resource for integration of soybean translational genomics and molecular breeding

**DOI:** 10.1093/nar/gkt905

**Published:** 2013-10-09

**Authors:** Trupti Joshi, Michael R. Fitzpatrick, Shiyuan Chen, Yang Liu, Hongxin Zhang, Ryan Z. Endacott, Eric C. Gaudiello, Gary Stacey, Henry T. Nguyen, Dong Xu

**Affiliations:** ^1^Department of Computer Science, University of Missouri, Columbia, MO 65211, USA, ^2^Christopher S. Bond Life Sciences Center, University of Missouri, Columbia, MO 65211, USA, ^3^National Center for Soybean Biotechnology, University of Missouri, Columbia, MO 65211, USA, ^4^Informatics Institute, University of Missouri, Columbia, MO 65211, USA and ^5^Division of Plant Sciences, University of Missouri, Columbia, MO 65211, USA

## Abstract

Soybean Knowledge Base (http://soykb.org) is a comprehensive web resource developed for bridging soybean translational genomics and molecular breeding research. It provides information for six entities including genes/proteins, microRNAs/sRNAs, metabolites, single nucleotide polymorphisms, plant introduction lines and traits. It also incorporates many multi-omics datasets including transcriptomics, proteomics, metabolomics and molecular breeding data, such as quantitative trait loci, traits and germplasm information. Soybean Knowledge Base has a new suite of tools such as *In Silico* Breeding Program for soybean breeding, which includes a graphical chromosome visualizer for ease of navigation. It integrates quantitative trait loci, traits and germplasm information along with genomic variation data, such as single nucleotide polymorphisms, insertions, deletions and genome-wide association studies data, from multiple soybean cultivars and *Glycine soja*.

## INTRODUCTION

Many genome-scale data are available in soybean (*Glycine max*) (1) including genomics, transcriptomics, proteomics and metabolomics datasets, together with growing knowledge of soybean genes, microRNAs (miRNAs), pathways and phenotypes. This represents a rich resource, which can provide valuable insights and better soybeans if mined in an innovative and integrative manner. Integrated molecular breeding and genomic approaches can lead to development of superior soybean genotypes with various desirable traits such as drought tolerance, pest resistance and higher nutritional qualities. This can be achieved by innovative and comprehensive informatics approaches that leverage from infrastructures built for genomics and multi-omics studies in soybean. While some resources currently available for soybean such as SoyBase (2), Soybean Genome Database (3) and Soybean Functional Genomics Database (http://bioinformatics.cau.edu.cn/SFGD/) provide partial solutions to these issues, a more comprehensive platform for integrating functional genomics and molecular breeding is needed. Soybean Knowledge Base (SoyKB) (4) provides an all-inclusive one-stop-shop resource for the soybean researcher, farmers and breeders to use such informatics approaches directly. SoyKB is publicly accessible at http://soykb.org. This update outlines the recent developments in SoyKB that have expanded its scope and applications to molecular breeding and translational genomics for soybean development.

Some of the key features developed include the addition of two new data entities, i.e. plant introduction (PI) lines and traits along with the *In Silico* Breeding Program, which include a suite of tools to integrate the translational genomics and molecular breeding data. Quantitative trait loci (QTL) for multiple traits can be queried and visualized in the chromosome visualizer simultaneously and overlaid on top of the genes and other molecular markers as well as multi-omics experimental data for meaningful inferences. It also has the germplasm (http://www.ars-grin.gov/npgs/index.html) information pages for 19 000 PIs as well as individual trait card pages. SoyKB has many new data analysis and visualization tools for RNA-seq and proteomics expression datasets including heatmaps, scatter plots and hierarchical clustering. It also has suite of tools for differential analysis of omics datasets. Various new types of data including DNA methylation, fast neutron mutations, phosphorylation, genotype by sequencing (GBS) data for molecular breeding and phenotypic inferences have been incorporated.

## NEW SOYKB FEATURES AND DATA ENTITIES

### SoyKB powered by iPlant Cyber-Infrastructure

SoyKB is now powered by the iPlant (5) Cyber-Infrastructure. The Web site is hosted on the iPlant’s advanced computing infrastructure established to leverage the data analysis capabilities. Supplementary Figure S1 shows a list of all data entities and analysis tools currently available in SoyKB for users. In the future, users will have access to many automated analysis pipelines for data processing and analyses using iPlant’s computational capabilities.

### SoyKB user groups and data sharing capabilities

#### SoyKB account registration

Users can register for a SoyKB account by visiting the sign-up page and filling in the required information. SoyKB currently has hundreds of registered users from all parts of the world and from both academia and industry. The advanced users have the privilege to add and share comments with other users.

#### Creation of groups

A newly developed feature in SoyKB allows the users to create their own groups. Users can view the details of the group and manage the group memberships by adding or deleting users. The creators of the group can also invite other users to the group by performing a search for registered users using name and institution keyword searches. An automatic email is sent by SoyKB system to the users and, once accepted, the users are added to the group. The group creator also has permission to edit or delete the group.

#### Sharing data with group members

Users can bring in their private data and share the data with the members belonging to their own or other groups. Access to the shared data in SoyKB is controlled based on group access privileges and the ownership of the datasets.

### New data entities in SoyKB

SoyKB has a dedicated entity card page containing all information associated with an individual entity in the database. SoyKB currently supports data for six entities including genes/proteins, miRNAs/small RNAs (sRNAs), metabolites, single nucleotide polymorphisms (SNPs), PIs lines and traits. The last two entities, as described later, are the most recent additions to SoyKB for supporting molecular breeding analysis.

#### PI line

The PI card includes information about the PI line number along with the quantitative and qualitative values of 150 descriptors falling in the categories of chemical descriptors, disease descriptors, growth descriptors, insect descriptors, morphology descriptors, phenology descriptors, qualifier and stress descriptors and other descriptors (Supplementary Figure S2). SoyKB currently stores information for all 19 000 PI lines from the USDA–ARS germplasm dataset. It also has many metabolic phenotypic data generated by our collaborators. The trait information and genomic variations from the SNP array as well as GBS resequencing data for respective PI lines have been incorporated for browsing on the PI card pages for individual chromosomes.

#### Traits

The trait card pages include information about the trait name and various QTL regions identified on each of the 20 chromosomes along with genes overlapping in individual QTL regions, and they are color coded based on individual QTL regions. It includes information for SNPs, insertions and deletions from 31 soybean genotypes (6) and *Glycine soja* (7) datasets as seen in Supplementary Figure S3. The PI lines known to be associated with certain traits are also listed on the trait card pages.

## *IN SILICO* BREEDING PROGRAM SUITE OF TOOLS

We have developed a suite of tools in the *In Silico* Breeding Program mainly for molecular breeders to allow the integration of the genomic variations alongside traits, QTL, germplasm datasets and genomic information. Many types of variation datasets such as SNPs and genome-wide association studies data from various soybean cultivars and *G. soja* have also been used for comparisons. The *In Silico* Breeding Program has four main menus as described later.

### Germplasm Browser

**Figure 1.**
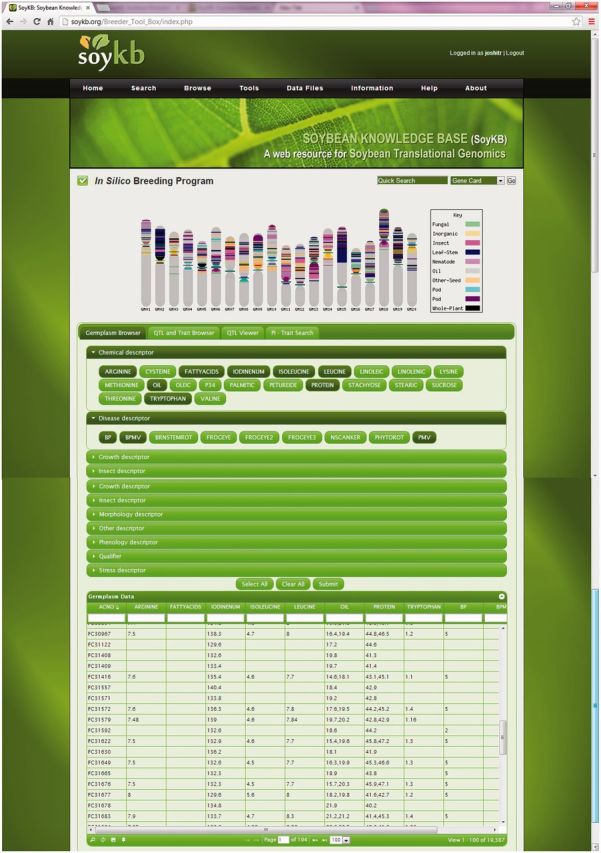
Germplasm Browser of the *In Silico* Breeding Program suite of tools showing phenotypic descriptors for ∼19 000 PI lines.

The Germplasm Browser uses the data generated by USDA–ARS (http://www.ars-grin.gov/npgs/index.html) and allows users to filter traits based on the combination of descriptors of interest or simply querying the entire dataset by selecting all descriptors. The phenotypic data has been classified into 150 descriptors as described earlier and can also be combined with the private phenotypic datasets generated by registered users. The results are presented in the form of a table, which can be searched by values, and sorted and filtered by using available features as shown in Figure 1. The results can also be exported in the form of a comma-separated values (CSV) file either before or after filtering. Users or groups can bring in their own private data for other phenotypic descriptors and access them combined with the public dataset in the Germplasm Browser tool.

### QTL and Trait browser

The QTL and Trait browser provide access to the traits information and overlay the information with the QTL, markers and overlapping genes together with the expression data from multi-omics experiments. Single or multiple traits can be selected and searched by selecting a chromosome of choice as shown in Supplementary Figure S4. The display shows the linkage group and chromosome coordinates along with overlapping genes and marker positions for the selected traits in the tabular format. The results can be filtered, sorted and also exported as a CSV file. For the overlapping genes, their functional annotation pie charts for gene families, PFAM (8), PANTHER (9) and KOG (10) annotations, and heatmaps for multi-omics expression datasets such as transcriptomics RNA-seq, microarray and proteomics are generated in the same tool for browsing.

### QTL viewer

**Figure 2.**
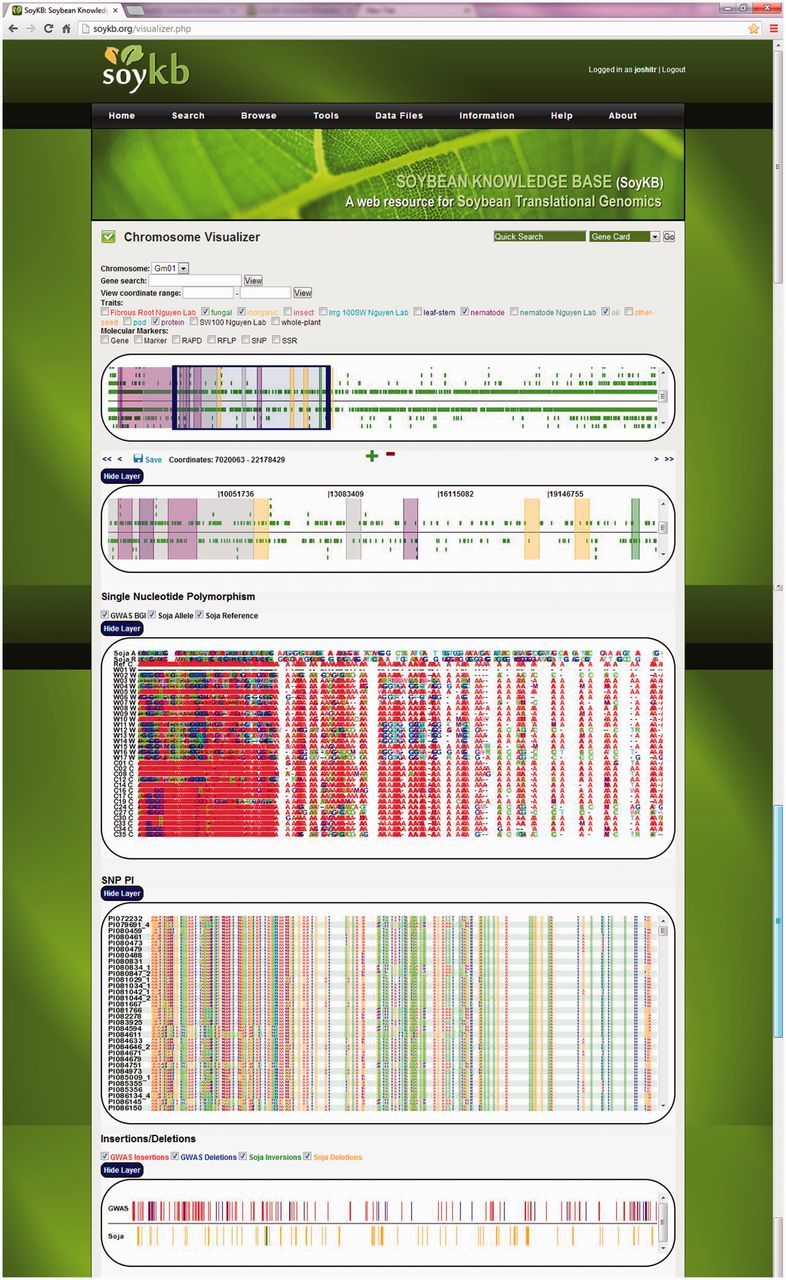
Example of QTL viewer in the *In Silico* Breeding Program suite of tools showing multiple traits in Gm01 along with underlying genes, GBS, SNP array and insertions/deletions data on the bottom panels, respectively.

The QTL viewer embodies an in-house-developed graphical chromosome visualizer displaying the analyzed data linearly for each chromosome along with other useful information such as QTL for various traits and SNPs from multiple genotypes. The chromosome visualizer has multiple panels, each representing a different type of available data such as QTL for various traits, genes underlying the selected regions on the chromosome, SNPs from GBS data, SNP array information and insertions and deletions. It can select multiple traits simultaneously, and each QTL region is color coded and coordinated with the same color used to represent the corresponding trait, thereby making it easier for users to distinguish various traits and QTL. The chromosome region can also be selected by chromosome coordinates or by entering a gene name to directly drill down to a specific region. Figure 2 shows an example of QTL in chromosome Gm01 with the oil, protein, inorganic, nematode and fungal traits highlighted in chromosome visualizer. Additional details describing the data panels are available in Supplementary Figure S5. The genomic variation SNP data from 31 soybean genotypes (6), as well as *G. soja* (7), are displayed on the SNPs and insertion/deletion panel. The ∼50 K markers SNP array data (11) for ∼1000 soybean lines can also be viewed on the SNP array panel.

### PI and trait search

The PI and trait search give easy access to the PI lines and traits. Multiple PI lines can be searched at one time, and results are presented in a tabular format for quick comparison. Single PI and trait searches pull out the exact same PI and trait card pages as described earlier.

## NEW DATA FOR BROWSING IN SOYKB

### DNA methylation data

The DNA methylation datasets generated for four genotypes, LD, LDX, 11 268 and 11 272 control and PAMP treatments (12), have also been incorporated in SoyKB on the individual gene card pages.

### Phosphorylation data

The phosphorylation sites for soybean proteins were identified using our in-house Plant Protein Phosphorylation Database (13). All the predicted phosphorylation sites are linked through SoyKB to the Plant Protein Phosphorylation Database Web site. The link gives details for phosphosites and also marks the phosphorylated positions in red.

### *G. soja* comparison with *G. max*

The comparison of *G. soja* with *G. max* (14) was conducted using published genomics sequences from both species. These results are made available to the users and integrated seamlessly with other data in SoyKB.

### Fast neutron mutant data

The fast neutron mutant data (15) generated from comparative genome hybridization (CGH) mutation studies in soybean are also available for browsing in SoyKB. The data can be queried using the CGH-ID, GlymaID, confidence levels and regions of a chromosome. Each query shows the information related to fast neutron study including the picture of the phenotype of the plant, CGH expression array data for all 20 chromosomes as log2 ratio and for any genes falling in this mutated region.

### Differential expression analysis

The differential expression analysis results for multi-omics experimental datasets can be browsed in SoyKB. The datasets can belong to any of the cDNA, oligo array, microarray, transcriptomics RNA-seq, proteomics or metabolomics experimental types, and results can be filtered based on fold change and *P*-value. The differential expression analysis suite of tools has five menus. (i) The Gene Lists menu gives access to the entire list of differential expression genes/proteins for all selected conditions. Currently, users can download the entire list of up- and downregulated gene lists or browse in a tabular format with every gene linked to its gene card page. (ii) The Venn diagram menu allows users to view the intersection of differential expressed genes between selected conditions as a graphical Venn diagram (Supplementary Figure S6) and also save the genes common between multiple conditions. (iii) The Volcano Plot menu gives users access to the volcano plot for both up- and downregulated genes along with log2 fold change and *P*-value (iv-v). The Function Analysis and Pathway Analysis menus give users access to the functional annotation pie charts for gene families, PFAM, PANTHER and KOG as well as a list of pathways the genes belong to, respectively.

## NEW TOOLS IN SOYKB

### Multiple sequence similarity

SoyKB also supports multiple sequence similarity searches for users by allowing them to upload gene lists and automatically fetch the sequences or directly upload a file with multiple sequences. It uses Clustal-Omega (16) for alignments and displays the alignment results with the option for users to download the results as a text file. Users can also perform phylogeny tree construction directly from the results.

### Phylogeny

The Phylogeny tool allows the users to start with a gene list and fetch the coding sequence (CDS), cDNA or protein sequence automatically from the database, select the clustering method for phylogenetic tree construction from neighbor-joining method or unweighted pair group method with arithmetic mean (UPGMA) and generate a phylogeny tree for multiple sequences.

### Protein BioViewer

**Figure 3.**
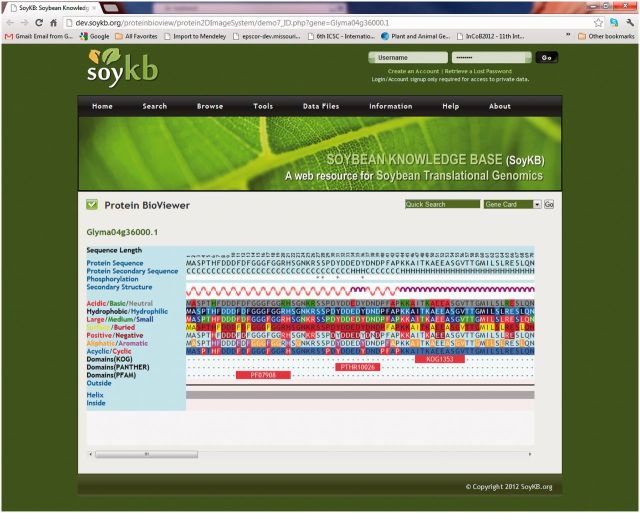
Protein BioViewer for protein Glyma04g36000 showing the primary sequence, secondary structures, amino acid characteristics and domain positions.

The Protein BioViewer is an in-house tool developed using CSS, HTML5 and PHP graphics, and it was designed to show the primary protein sequence, predicted secondary structures, phosphorylation positions, amino acid characteristics, functional domain positions and trans-membrane helix positions. The functional domains have been predicted using InterPro Scan (17), which includes domains such as from PFAM, PANTHER, SUPERFAMILY, PRINTS, SMART, GENE3D, TIGERFAMs, PIR, SEG and COIL. Each domain is linked to its own Web site for some domain categories, and users can click on the domain to go directly to the corresponding Web sites for more details. Figure 3 shows the visualization of protein Glyma04g36000 in the Protein BioViewer.

### Heatmap and hierarchical clustering

**Figure 4.**
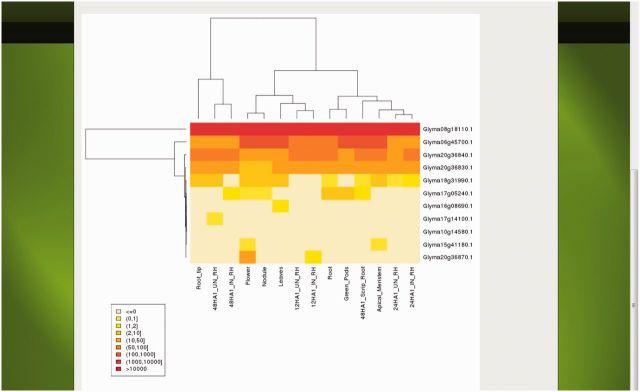
Heatmap and hierarchical clustering tool for viewing RNA-seq expression data for multiple genes.

Expression data for multiple genes or proteins can be viewed in the heatmap and hierarchical clustering tool for the transcriptomics RNA-seq, microarray, cDNA array, oligo array and proteomics data types. Users can input a list of genes or proteins, which enables them to select a dataset and the conditions to view from transcriptomics or proteomics data types. The data are clustered based on the gene list entered and the data samples selected to generate a hierarchical clustering. The color scale of the heatmaps is automatically scaled as per the datasets and divided into suitable intervals. Figure 4 shows the heatmap for multiple genes generated for the RNA-seq dataset.

### Scatter plots

The scatter plot tool displays expression data from two gene expression datasets at a time. This tool is currently available only for the transcriptomics datasets, and it can be used to plot one sample against the other irrespective of whether they are replicates or different conditions. The tool also calculates the equation and *R*^2^ regression value based on selected datasets. A distribution along the diagonal for replicates shows less variability, whereas a deviation away from the diagonal shows differentially expressed genes when comparing the two separate conditions.

### New FTP/data download capabilities

SoyKB now also provides various FTP and data download capacities for its users. Next generation sequencing (NGS) raw read sequencing files are also provided for certain NGS experiments through the Data Files menu.

## FUTURE DIRECTIONS

SoyKB web resource is under active development with new datasets or analysis tools being constantly added to address all aspects of soybean research. We are currently developing new tools such as KBCommons system, Genotype2Phenotype and Cyber Studio system. The KBCommons system will allow quick and easy replication of the basic architecture for the SoyKB system for other biological species. The basic information such as genomics sequences, gene model annotations, functional annotations, experimental data (e.g. transcriptomics, proteomics and metabolomics data) and genomic variations data (e.g. SNPs and insertion/deletions) can be quickly integrated using standardized entity structures. The Genotype2Phenotype prediction tool will provide the results of computational predictions using the LASSO and Elastic Net methods in identifying the most informative SNPs for a specific phenotype or trait. The methods use the SNP array or GBS data for PI lines and corresponding phenotypic information and predict the ranking of the top most informative SNPs, which can differentiate between the phenotypic data and explain the differences. It also addresses the SNP–SNP interaction between multiple associated SNPs. The results will be seamlessly integrated with the other data in SoyKB and a list of QTL and traits and genes that overlap with the identified significant SNPs. The Cyber Studio system is also currently being developed as a web-based tool designed to allow biologists and researchers to use the multi-omics datasets in SoyKB and build draft modules to generate or validate *in silico* hypothesis. It will include incorporation of differentially expressed genes identified from microarray or RNA-seq data, proteins, metabolites and miRNA in the hypothesis generation.

## SUPPLEMENTARY DATA

Supplementary Data are available at NAR Online.

## FUNDING

The iPlant Collaborative (www.iplantcollaborative.org) is funded by a grant from the National Science Foundation [#DBI-0735191]; The Missouri Soybean Merchandising Council [MSMC #306 to D.X., H.T.N., G.S.]; United Soybean Board [project 8236 to H.N., G.S. and D.X.]; National Science Foundation [#DBI-0421620 to G.S. and D.X.]; Department of Energy [DE-SC0004898 to G.S., D.X.]; National Center for Soybean Biotechnology. Funding for open access charge: National Science Foundation [#DBI-0421620].

*Conflict of interest statement*. None declared.
